# Molecular Sex Differences in Human Serum

**DOI:** 10.1371/journal.pone.0051504

**Published:** 2012-12-12

**Authors:** Jordan M. Ramsey, Emanuel Schwarz, Paul C. Guest, Nico J. M. van Beveren, F. Markus Leweke, Matthias Rothermundt, Bernhard Bogerts, Johann Steiner, Liliana Ruta, Simon Baron-Cohen, Sabine Bahn

**Affiliations:** 1 Department of Chemical Engineering and Biotechnology, University of Cambridge, Cambridge, United Kingdom; 2 Department of Psychiatry, Erasmus University Medical Center, Rotterdam, The Netherlands; 3 Department of Psychiatry and Psychotherapy, University of Cologne, Cologne, Germany; 4 Central Institute of Mental Health, University of Heidelberg, Mannheim, Germany; 5 Department of Psychiatry, University of Muenster, Muenster, Germany; 6 Department of Psychiatry, University of Magdeburg, Magdeburg, Germany; 7 Autism Research Centre, Department of Psychiatry, University of Cambridge, Cambridge, United Kingdom; 8 Department of Neuroscience, Erasmus University Medical Centre, Rotterdam, The Netherlands; Aligarh Muslim University, India

## Abstract

**Background:**

Sex is an important factor in the prevalence, incidence, progression, and response to treatment of many medical conditions, including autoimmune and cardiovascular diseases and psychiatric conditions. Identification of molecular differences between typical males and females can provide a valuable basis for exploring conditions differentially affected by sex.

**Methodology/Principal Findings:**

Using multiplexed immunoassays, we analyzed 174 serum molecules in 9 independent cohorts of typical individuals, comprising 196 males and 196 females. Sex differences in analyte levels were quantified using a meta-analysis approach and put into biological context using k-means to generate clusters of analytes with distinct biological functions. Natural sex differences were established in these analyte groups and these were applied to illustrate sexually dimorphic analyte expression in a cohort of 22 males and 22 females with Asperger syndrome. Reproducible sex differences were found in the levels of 77 analytes in serum of typical controls, and these comprised clusters of molecules enriched with distinct biological functions. Analytes involved in fatty acid oxidation/hormone regulation, immune cell growth and activation, and cell death were found at higher levels in females, and analytes involved in immune cell chemotaxis and other indistinct functions were higher in males. Comparison of these naturally occurring sex differences against a cohort of people with Asperger syndrome indicated that a cluster of analytes that had functions related to fatty acid oxidation/hormone regulation was associated with sex and the occurrence of this condition.

**Conclusions/Significance:**

Sex-specific molecular differences were detected in serum of typical controls and these were reproducible across independent cohorts. This study extends current knowledge of sex differences in biological functions involved in metabolism and immune function. Deviations from typical sex differences were found in a cluster of molecules in Asperger syndrome. These findings illustrate the importance of investigating the influence of sex on medical conditions.

## Introduction

Sexual dimorphism in underlying processes in medical conditions are numerous and diverse, occurring in diseases ranging from autoimmune and cardiovascular conditions to neurological conditions [1]–[3]. Parameters such as disease prevalence, incidence, age at onset, progression, mortality, and treatment response can show sex differences [4]. The key to some of these differences may be in sex-dependent regulation of biological pathways. Such differences have been investigated in the context of immune response, and elevated immune activation in females has been linked to the significantly increased susceptibility of women to multiple sclerosis (MS) and other autoimmune diseases such as rheumatoid arthritis, Grave’s disease, and lupus erythematosus [1]. Several possible avenues have been explored to explain such differences in autoimmune diseases. For example, one study showed that typical female mice had detectable levels of immunoglobulin G auto-antibodies that were absent in male mice [5]. Similarly, higher blood immunoglobulin levels and CD4/CD8 T-cell ratios have been found in typical women, along with lower natural killer cell and antibody-dependent cell-mediated cytotoxicity [1]. Sex dimorphic expression of particular cytokines, such as transforming growth factor (TGF)-β1 and interleukin (IL)- 4, has also been implicated in osteoarthritis in mice [6].

Physiological and molecular mechanisms causing sex dimorphisms have also been investigated in the context of cardiovascular diseases. Myocardial infarctions in women result in a higher mortality rate and poorer prognosis compared to men [7]. Reports have shown that sex differences in myocardial function appear during physiological stress [7]. Sex differences in the stress responses of rodent cells have been observed along with sexually dimorphic gene expression of stress-related genes both before and after application of stress [8]. This study showed intrinsic sex differences in cell response to stressors such as ethanol and influenza A virus even without exposure to sex hormones [8]. Basal hypothalamic-pituitary-adrenal (HPA) axis function was also increased in females [9]. It has been suggested that the reproductive and HPA axes work together with the immune system to maintain homeostasis [9]. Stress and immune markers are likely to show sex specific expression and response to stimuli, potentially creating different susceptibilities to autoimmune, cardiovascular, and other diseases in which stress and immune responses play a role in disease vulnerability.

An increased understanding of sex dimorphisms in biological regulation may also help to elucidate potential differences in treatment response. For example, growth hormone, which has been used to treat a variety of irregularities associated with cardiovascular, immune, metabolic, psychological and other biological functions, is less effective in women than men [10]. Sex differences in growth hormone regulation were found to be responsible for these differences. The actions of growth hormone, whose release is continuous in females and episodic in males, were suppressed in cells from females [10]. This suppression has been hypothesized to be due to suppressed signalling through cytokines and the Jak2/Stat5B pathway that usually activates growth hormone [10].

Studies measuring sex differences at the molecular level have so far been limited to the investigation of only a few molecules and specific disease and inflammation processes [1], [11]–[14]. The aim of the present study was to elucidate such differences at a systematic level through measurement of 174 serum molecules in a large cohort of typical individuals. The investigated molecules included cytokines, chemokines, hormones, growth factors, angiogenesis and central nervous system-related analytes, as well as other serum proteins important in disease (Myriad RBM website. Available: http://myriadrbm.com/. Accessed 2012 May 18). The applied multiplexed immunoassay platform has been used previously to explore molecular changes in cancer and autoimmune, cardiovascular, gastrointestinal, neurological, and various other diseases, many of which show sex differences [15]–[20]. In addition, we attempted to relate the discovered sex differences to those measured in samples from participants with Asperger syndrome. This condition shows a particularly pronounced sex dimorphism as the prevalence is 4–10-fold higher in males [21].

## Materials and Methods

### Clinical Samples

Protocols for the study were approved by ethical committees at all involved university hospitals (see below) and carried out in accordance with the Declaration of Helsinki. Informed written consent was obtained from all participants. Individuals with a family history of mental illness or medical conditions like hypertension, type II diabetes, cardiovascular, or autoimmune diseases were excluded from the study. Pregnant females were also excluded from the study. A total of nine cohorts of typical individuals were used in this investigation. Cohort 1 was from the Autism Research Centre, University of Cambridge, Department of Psychiatry, UK; cohorts 2 and 3 were from University of Cologne Department of Psychiatry, Germany; cohorts 4 to 6 were from the University of Muenster, Germany; cohorts 7 and 8 were from the University of Magdeburg, Germany; and cohort 9 was from Erasmus Medical Centre in the Netherlands. In addition, a separate cohort of adults with Asperger syndrome (Cohort 10) was obtained from the Autism Research Centre, University of Cambridge, Department of Psychiatry, UK. Diagnosis of Asperger syndrome by psychiatrists was based on the Structured Clinical Interview for the Diagnostic and Statistic Manual-IV-Text Review Disorders and the DSM-IV-TR. Biological sex was used to classify males and females. Females were not excluded for contraceptive use and were at variable times in the menstrual cycle. Blood was collected in the morning after overnight fasting, serum prepared and 181–247 analytes measured using multiplexed immunoassay analyses in a CLIA-certified laboratory at Myriad-RBM (Austin, TX, USA), as described previously [18].

### Data Pre-processing

The statistical programming software R was used to match controls, replace missing analyte values, remove outliers, and to perform meta-analysis. For each of the nine cohorts, typical males and females were matched according to age, BMI, waist circumference, smoking, and cannabis consumption. Demographic details including available metadata used for matching in all nine cohorts are shown in **Table 1**. Values outside the linear range of the assay system were either replaced by half the minimum value or by double the maximum value measured for the respective analyte. Analytes with more than 70% missing values were eliminated from the dataset. Outlier removal was performed for each analyte separately and values more than four standard deviations from the overall mean were excluded.

**Table 1:** Metadata for all participants.

	Cohort1	Cohort2	Cohort3	Cohort4	Cohort5	Cohort6	Cohort7	Cohort8	Cohort9	Cohort 10 (AS)
Controls	40	50	14	20	26	22	162	44	14	44
Controls (M/F)	20/20	25/25	7/7	10/10	13/13	11/11	81/81	22/22	7/7	22/22
Age (M)	32±8	29±7	38±9	24±7	32±11	38±11	37±11	34±11	27±2	30±9
Age (F)	32±6	30±7	38±9	24±7	32±13	39±12	37±12	37±12	28±2	33±8
Age Range (M)	19–45	19–44	29–52	18–42	20–56	20–61	19–60	19–55	25–29	19–43
Age Range (F)	20–42	21–44	28–50	18–43	20–61	20–61	18–62	21–52	25–30	18–45
BMI (M)		23±2					26±4	24±2		
BMI (F)		22±4					25±4	23±3		
WC (M)							102±8	96±6		
WC (F)							103±11	96±6		
Smoking (M) (Y/N/NA)	3/17/0	11/14/0	0/7/0				12/69/0	6/16/0		5/17/0
Smoking (F) (Y/N/NA)	3/17/0	12/13/0	1/6/0				17/64/0	3/19/0		5/17/0
Cannabis (M) (Y/N/NA)	0/17/3	13/11/1	0/7/0				0/81/0	0/22/0		2/14/6
Cannabis (F) (Y/N/NA)	1/14/5	14/10/1	1/5/1				0/81/0	0/22/0		0/6/16

Nine cohorts of typical individuals and one cohort of individuals with Asperger syndrome were recruited. Values are reported as mean ± standard deviation. BMI (body mass index, in kg/m^2^), WC (waist circumference, in centimetres).

### Meta-analysis

Meta-analysis was carried out using the non-parametric Cliff’s delta as a measure of effect size [22]–[25]. Cliff’s delta estimated the probability that the level of an analyte was higher in males than in females versus the reverse probability. Cliff’s delta was calculated in all cohorts to quantify the difference in molecular levels between males and females, and the pooled effect was determined using a random effects meta-analysis. This approach was chosen after finding significant heterogeneity between cohorts as assessed by a standard χ^2^ test [26]. Random effects meta-analysis accounts for this heterogeneity allowing cohort-specific average effects to vary as part of a common distribution. This approach has been used recently to detect novel loci in genome wide association studies for various disorders [27], [28]. Other uses have ranged from assessing the effect of intentional weight loss on depressive symptoms to examining the relationship between physical activity and risk of colon adenoma [29], [30]. For the determination of the pooled effect size, a minimum of three cohorts was required for each analyte.

All determined p-values were adjusted for the false discovery rate according to the method of Benjamini and Hochberg [31]. Adjusted p-values (q-values) of less than 0.05 were considered to indicate statistical significance.

### K-means Clustering and Principal Component Analysis

To assign molecules to biological pathways, we first employed a clustering approach to group analytes with similar concentration patterns. For this purpose, k-means clustering was performed on standardized analyte levels measured in the largest cohort (cohort 7). To avoid the influence of outlying observations on the clustering, such values were replaced with a uniform random number between the minimum and maximum analyte values. The number of clusters was pre-specified to 6, but different qualitatively similar results were obtained by using other cluster numbers.

The main biological functions for the analytes in each cluster of related molecules were identified using Ingenuity Pathway Knowledgebase (IPA) software with all measured molecules as the reference dataset (Ingenuity website. Available: http://www.ingenuity.com/. Accessed 2012 April 18). Main biological functions were determined by identifying overrepresented functions of the molecules in the cluster. These biological functions were verified using the Database for Annotation, Visualization and Integrated Discovery (DAVID ) version 6.7 (DAVID website. Available: http://david.abcc.ncifcrf.gov/. Accessed 2012 Oct 16) [32], [33]. The IPA software was also used to create networks for clustered analytes, taking into consideration direct and indirect relationships between proteins.

The molecular clusters and their relationships to results from the meta-analysis of sex differences were visualized using principal component analysis (PCA; SIMCA-P+ Version 12.0). Principal components were used to create a single composite variable summarizing the information of all molecules in each cluster. For this purpose, we used the first principal component of standardized analyte concentrations in each cluster and determined sex differences. These were combined across cohorts using a random effects meta-analysis. Since principal components are indifferent with respect to sign, this was determined using the average sign of the individual standardized molecular concentrations within each cluster. The average sign was calculated from the molecules with the highest principal component loadings. Subsequently, ANOVA was used on all principal component scores to quantify sex-by-condition interactions in groups of analytes. P-values were adjusted for the false discovery rate with the Benjamini and Hochberg method [31].

## Results

### Meta-analysis

After data pre-processing, a pooled Cliff’s delta was calculated for each of the 174 serum molecules across the nine cohorts of typical individuals. Of these, a total of 77 analytes were present at significantly different concentrations between male and female participants after adjusting for the false discovery rate (q <0.05, **Table 2** **and** **3**). A positive Cliff’s delta value indicates that a given analyte was higher in males more often, and a negative Cliff’s delta value indicates the reverse. **Table 2** shows that 40 molecules had higher concentrations in females and **Table 3** shows that 37 molecules were more frequently higher in male participants. Most data for the displayed analytes had no missing values and all but five had less than 15% missing values.

**Table 2:** Analytes significantly elevated in females.

Energy production & fatty acid oxidation/hormone levels	Cell death	Immune cell chemotaxis	Other	Immune cell growth/activation	Lipid transport/homeostasis	Analyte	UniProt	Cliff's δ (95% CI)	q-value	Mean (m/f)
✓						Growth Hormone	P01241	−0.86 (−0.95, −0.77)	<0.001	0.6/6.3 ng/mL
✓						Sex Hormone Binding Globulin	P04278	−0.81 (−0.91, −0.70)	<0.001	35.4/126.7 nmol/L
✓						Trefoil Factor 3	Q07654	−0.76 (−0.90, −0.63)	<0.001	0.1/0.4 ug/mL
✓						Leptin	P41159	−0.75 (−0.83, −0.68)	<0.001	4.5/13.7 ng/mL
✓						Apolipoprotein AI	P04639	−0.63 (−0.76, −0.49)	<0.001	0.5/0.7 mg/mL
✓						Adiponectin	Q15848	−0.58 (−0.72, −0.44)	<0.001	3.5/5 ug/mL
✓						Thyroxine Binding Globulin	P05543	−0.51 (−0.62, −0.40)	<0.001	50.7/65.4 ug/mL
✓						N-terminal Prohormone of Brain Natriuretic Peptide	P16860	−0.50 (−0.72, −0.28)	<0.001	295.5/503.1 pg/mL
✓						Alpha 2 Macroglobulin	P01023	−0.49 (−0.60, −0.38)	<0.001	1/1.3 mg/mL
✓						Alpha 1 Antitrypsin	P01009	−0.42 (−0.60, −0.24)	<0.001	2/2.5 mg/mL
✓						Epithelial Derived Neutrophil Activating Protein 78	P42830	−0.40 (−0.51, −0.28)	<0.001	1.6/2.3 ng/mL
✓						C-Reactive Protein	P02741	−0.36 (−0.53, −0.19)	<0.001	1/1.6 ug/mL
✓						Stem Cell Factor	P21583	−0.28 (−0.40, −0.15)	<0.001	355.6/429.7 pg/mL
✓						B Lymphocyte Chemoattractant	O43927	−0.24 (−0.37, −0.12)	<0.001	15.6/23.7 pg/mL
✓						Angiopoietin 2	O15123	−0.22 (−0.35, −0.08)	0.001	1.9/2.3 ng/mL
✓						Receptor for Advanced Glycosylation End Products	Q15109	−0.15 (−0.28, −0.01)	0.039	4.3/4.9 ng/mL
	✓					Matrix Metalloproteinase 9 Total	P14780	−0.34 (−0.55, −0.13)	0.001	626.6/641.4 ng/mL
	✓					Immunoglobulin M	N/A	−0.31 (−0.47, −0.14)	<0.001	1.8/2.1 mg/mL
	✓					Lectin Like Oxidized Low-Density Lipoprotein (LDL) Receptor 1	P78380	−0.29 (−0.53, −0.05)	0.018	1.4/1.7 ng/mL
	✓					Tumour Necrosis Factor (TNF)-Related Apoptosis Inducing Ligand Receptor 3	O14798	−0.27 (−0.40, −0.14)	<0.001	10.3/11.4 ng/mL
	✓					Granulocyte Colony Stimulating Factor	P09919	−0.24 (−0.39, −0.09)	0.002	5.8/7.4 pg/mL
	✓					T Lymphocyte Secreted Protein I-309	P22362	−0.17 (−0.32, −0.03)	0.020	144.5/209.5 pg/mL
		✓				Tamm Horsfall Urinary Glycoprotein	P07911	−0.28 (−0.42, −0.13)	<0.001	0.055/0.059 ug/mL
		✓				Matrix Metalloproteinase 7	P09237	−0.27 (−0.47, −0.07)	0.008	3.2/3.7 ng/mL
		✓				Growth Regulated alpha Protein	P09341	−0.22 (−0.36, −0.09)	0.001	588/660.6 pg/mL
		✓				T Cell Specific Protein RANTES (acronym for Regulated upon Activation, Normal T-cell Expressed and presumably Secreted)	P30882	−0.18 (−0.34, −0.01)	0.035	17.3/20.2 ng/mL
			✓			Immunoglobulin A	N/A	−0.29 (−0.43, −0.15)	<0.001	2.9/2.6 mg/mL
				✓		Interleukin-3	P08700	−0.39 (−0.53, −0.24)	<0.001	0.05/0.065 ng/mL
				✓		Interleukin-5	P05113	−0.31 (−0.49, −0.12)	0.001	4.1/4.9 pg/mL
				✓		Interleukin-7	P13232	−0.29 (−0.41, −0.16)	<0.001	57.4/69.5 pg/mL
				✓		Thrombopoietin	P40225	−0.28 (−0.44, −0.11)	0.001	3/3.2 ng/mL
				✓		Interleukin-12 Subunit p40	P29460	−0.24 (−0.44, −0.04)	0.020	0.16/0.21 ng/mL
				✓		Interleukin-15	P40933	−0.23 (−0.36, −0.10)	0.001	0.37/0.45 ng/mL
					✓	Haptoglobin	P00738	−0.28 (−0.44, −0.12)	0.001	1.3/1.5 mg/mL
					✓	Fetuin A	P02765	−0.28 (−0.49, −0.07)	0.009	1126.5/1252.8 ug/mL
					✓	Apolipoprotein CIII	P02656	−0.23 (−0.36, −0.09)	0.001	100.7/114.1 ug/mL
					✓	Factor VII	P08709	−0.22 (−0.36, −0.07)	0.004	416.3/490.4 ng/mL
					✓	Vitronectin	P04004	−0.21 (−0.37, −0.05)	0.009	1434.2/1535.2 ug/mL
					✓	Serotransferrin	P02787	−0.21 (−0.38, −0.04)	0.014	2653.6/2813.7 mg/dl
					✓	Fibrinogen	P02671	−0.13 (−0.26, −0.01)	0.040	0.017/0.016 mg/mL

All analytes with a meta-analysis p-value of less than 0.05 after adjustment for the false discovery rate are shown. Analytes are ordered by biological function.

**Table 3:** Analytes significantly elevated in males.

Energy production & fatty acid oxidation/hormone levels	Cell death	Immune cell chemotaxis	Other	Immune cell growth/activation	Lipid transport/homeostasis	Analyte	UniProt	Cliff's δ (95% CI)	q-value	Mean (m/f)
		✓				Osteopontin	P10451	0.57 (0.36, 0.77)	<0.001	8.5/5.7 ng/mL
		✓				Thrombomodulin	P07204	0.45 (0.31, 0.59)	<0.001	5.1/4.4 ng/mL
		✓				AXL Receptor Tyrosine Kinase	P30530	0.45 (0.34, 0.56)	<0.001	11.9/9.5 ng/mL
		✓				Ferritin	P02792	0.41 (0.24, 0.59)	<0.001	89.1/43.3 ng/mL
		✓				Superoxide Dismutase 1 Soluble	P00441	0.40 (0.27, 0.53)	<0.001	26.1/20.9 ng/mL
		✓				Prostatic Acid Phosphatase	P15309	0.39 (0.26, 0.52)	<0.001	0.8/0.6 ng/mL
		✓				Eotaxin 1	P51671	0.38 (0.22, 0.54)	<0.001	213.4/151.3 pg/mL
		✓				Macrophage Inflammatory Protein 1 beta	P13236	0.37 (0.21, 0.53)	<0.001	245.3/200.9 pg/mL
		✓				Macrophage Inflammatory Protein 3 alpha	P78556	0.34 (0.19, 0.48)	<0.001	38.6/29.7 pg/mL
		✓				Fas Ligand Receptor	P25445	0.33 (0.21, 0.46)	<0.001	9.4/7.9 ng/mL
		✓				E-Selectin	P16581	0.31 (0.16, 0.45)	<0.001	548.8/426.5 ng/mL
		✓				Macrophage Inflammatory Protein 1 alpha	P10147	0.25 (0.09, 0.42)	0.003	81.6/73.7 pg/mL
		✓				Thymus Expressed Chemokine	O15444	0.25 (0.11, 0.40)	0.001	98.2/88 ng/mL
		✓				Epiregulin	O14944	0.24 (0.08, 0.41)	0.004	43.8/29.6 pg/mL
		✓				Chemokine CC-4	O15467	0.23 (0.04, 0.43)	0.017	4.1/3.6 ng/mL
		✓				Monocyte Chemotactic Protein 4	Q99616	0.23 (0.08, 0.39)	0.003	622/541.1 pg/mL
		✓				Monocyte Chemotactic Protein 1	P13500	0.22 (0.08, 0.36)	0.002	427.7/387.5 pg/mL
		✓				Thrombospondin 1	P07996	0.21 (0.03, 0.40)	0.023	43,837/39,513 ng/mL
		✓				Cystatin C	P01034	0.21 (0.05, 0.38)	0.011	983.6/927.2 ng/mL
		✓				Pulmonary and Activation Regulated Chemokine	P55774	0.17 (0.03, 0.31)	0.020	46.8/41.2 ng/mL
		✓				Vascular Cell Adhesion Molecule 1	P19320	0.15 (0.29, 0.004)	0.044	597.4/561.6 ng/mL
			✓			Prostate Specific Antigen Free	P07288	0.94 (0.79, 1.0)	<0.001	0.21/0.01 ng/mL
			✓			Matrix Metalloproteinase 3	P08254	0.91 (0.86, 0.97)	<0.001	8.7/4.3 ng/mL
			✓			Testosterone Total	N/A	0.90 (0.79, 1.0)	<0.001	4.9/1.2 ng/mL
			✓			Myoglobin	P02144	0.48 (0.37, 0.59)	<0.001	13/9.4 ng/mL
			✓			Creatine Kinase-Myocardial Band(MB)	P12277	0.43 (0.28, 0.57)	<0.001	1.2/0.8 ng/mL
			✓			Carcinoembryonic Antigen	P06731	0.33 (0.20, 0.45)	<0.001	1.1/0.8 ng/mL
			✓			Macrophage Colony Stimulating Factor 1	P09603	0.22 (0.07, 0.37)	0.004	0.082/0.084 ng/mL
			✓			Insulin like Growth Factor Binding Protein 2	P18065	0.20 (0.05, 0.34)	0.008	61.6/55.2 ng/mL
					✓	Vitamin K Dependent Protein S	P07225	0.42 (0.23, 0.61)	<0.001	23.8/20.1 ug/mL
					✓	Transthyretin	P02766	0.40 (0.27, 0.53)	<0.001	352.9/302.3 mg/dl
					✓	Serum Amyloid P Component	P02743	0.37 (0.23, 0.50)	<0.001	20/16.5 ug/mL
					✓	Alpha Fetoprotein	P02771	0.36 (0.21, 0.52)	<0.001	1.2/1 ng/mL
					✓	Apolipoprotein D	P05090	0.30 (0.09, 0.51)	0.006	158.2/133.8 ug/mL
					✓	Alpha 1 Microglobulin	P02760	0.29 (0.15, 0.43)	<0.001	9.8/9 ug/mL
					✓	Cluster of Differentiation (CD)5	P06127	0.28 (0.12, 0.44)	0.001	2280.8/2041 ng/mL
					✓	Apolipoprotein H	P02749	0.22 (0.06, 0.39)	0.008	263.4/244 ug/mL

All analytes with a meta-analysis p-value of less than 0.05 after adjustment for the false discovery rate are shown. Analytes are ordered by biological function.

### Clusters and Molecular Sex Differences

Functional assignment of analytes was performed by clustering molecular data from cohort 7 and determining the significantly enriched biological functions of the molecules in each cluster (**Table 2** **and** **3**). The full list of clustered molecules and their assignment to the main biological function as determined by IPA software can be found in **Table S1**. Apart from one, all clusters were assigned a distinct biological function.

**Figure 1:**
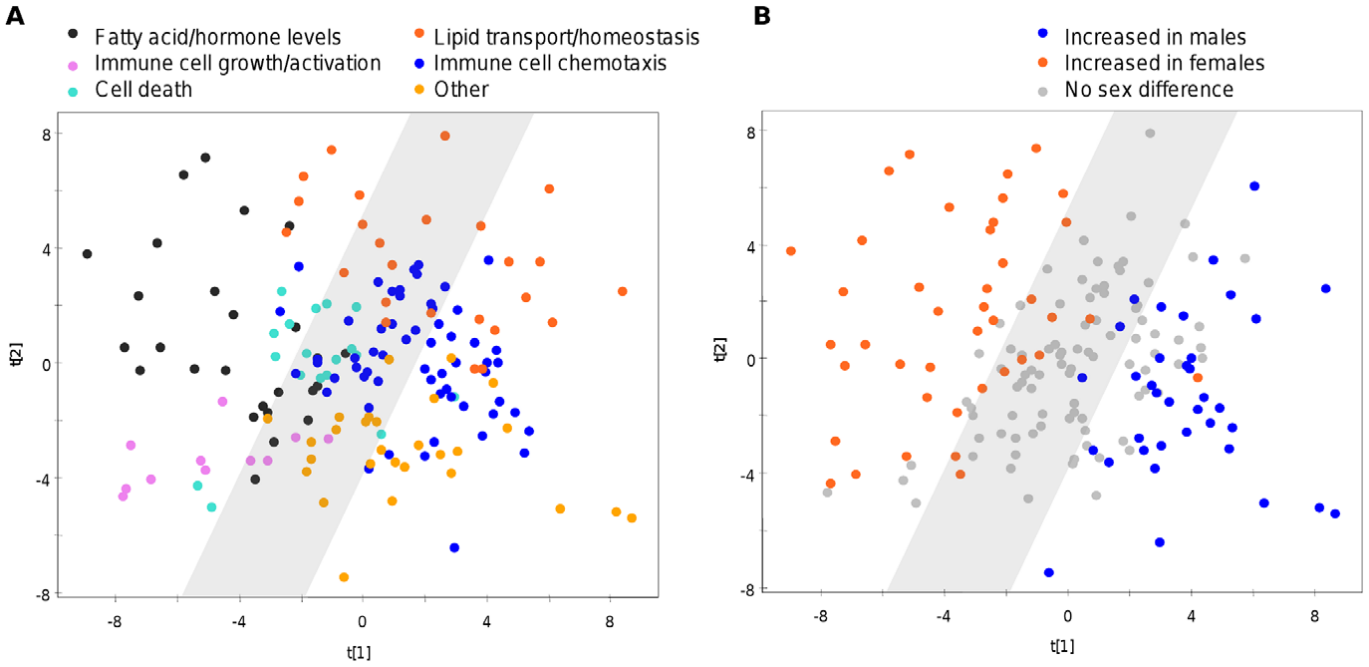
PCA plots showing individual molecules. A) Assignment of analyte clusters to biological functions; B) Sex differences as determined by meta-analysis. Colouring indicates significance (q<0.05). The grey area indicates analytes that show no significant sex differences. Plots were generated using data from cohort 7, in which 167 analytes were measured in 162 typical controls (81 females, 81 males).

Molecular clusters and their relationships to sex differences were visualized using PCA (**Figure 1A**). This figure reflects the difference in molecular profiles between clusters resulting from the applied clustering procedure. **Figure 1B** shows the same plot with identified molecular sex differences determined by the meta-analysis. The clustered groups with distinct biological functions mainly showed consistent sex differences in analyte levels.

**Figure 2:**
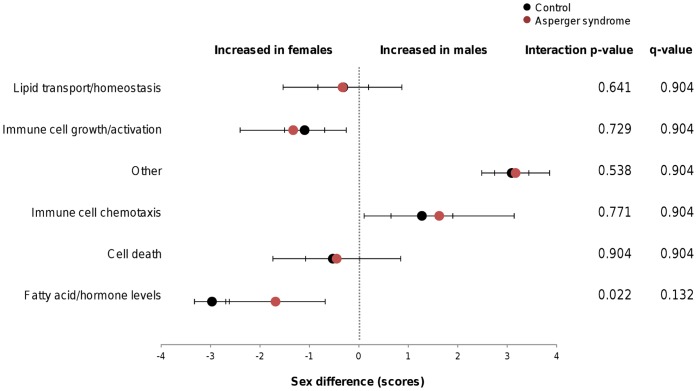
Sex differences of composite variables summarizing analyte clusters. Values for typical individuals were pooled across nine cohorts; values for Asperger syndrome participants were calculated from cohort 10. The x-axis shows the difference between composite values that reflect the average molecular levels in males and females. Horizontal bars indicate 95% confidence intervals of the difference between sexes.

We then assessed sex differences in each of the clustered groups of analytes combined across all cohorts using a composite value. This value summarized the sex differences observed in a given group of molecules (**Figure 2**) and mirrored the changes seen for individual analytes (**Figure 1**). The cluster associated with energy production, fatty acid metabolism, and hormone levels showed the most significant sex differences for groups with a distinct biological function. This included molecules such as growth hormone, sex hormone binding globulin, trefoil factor 3, leptin, apolipoprotein AI, adiponectin and thyroxine binding globulin. These all showed higher levels in females compared to males with ratios ranging from 1.4 to 10.0 (**Table 2**). The molecules which were more abundant in males were associated mainly with immune cell chemotaxis, although the ratiometric differences were smaller than those observed for catabolism of fatty acids and regulation of hormone levels in females (**Table 3**).

### Identification of Molecular Differences in Asperger Syndrome Associated with Sex

We next applied the sex differences identified in typical controls to investigate the potential association of these with Asperger syndrome. **Figure 2** shows the overlay of the naturally occurring sex differences found for each biological function with those observed in a cohort of 44 individuals with Asperger syndrome. Most sex differences were consistent with those observed in typical participants, further validating the result of the meta-analysis. However, a disease-sex interaction approaching significance was observed for molecules associated with fatty acid metabolism and hormone production (*P* = 0.022; q = 0.132) (**Figure 2**). This indicates that females with Asperger syndrome showed lower levels of these molecules than would be expected from the meta-analysis of typical controls.

**Figure 3:**
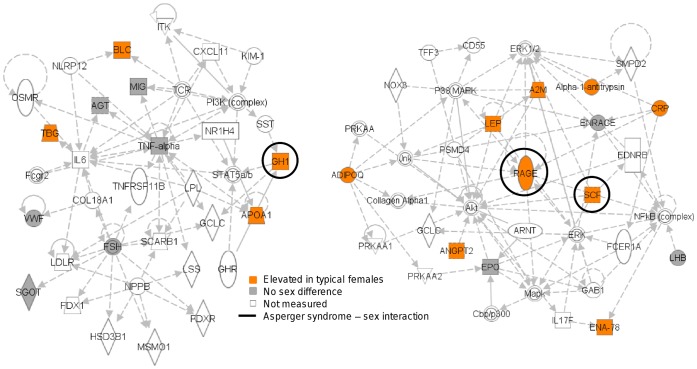
Sex-specific effects in networks for Asperger syndrome. Top networks of the cluster of molecules associated with energy production, fatty acid metabolism, and hormone levels from Ingenuity Pathway Knowledgebase software. Individual molecules are coloured according to significant sex difference in controls. Molecules with significant sex-disease interactions from [34] are circled. A2M (alpha 2 macroglobulin), ADIPOQ (adiponectin), RAGE (receptor for advanced glycosylation end products), ANGPT2 (angiopoietin 2), ARNT (aryl hydrocarbon receptor nuclear translocator), CRP (C-reactive protein), ENA-78 (epithelial derived neutrophil activating protein 78), EDNRB (endothelin receptor type B), EPO (erythropoietin), ERK (extracellular-signal-regulated kinase), FCER1A (Fc fragment of IgE, high affinity I, receptor for alpha polypeptide), GAB1 (GRB2-associated binding protein 1), GCLC (glutamate-cysteine ligase, catalytic subunit), IL17F (interleukin-17F), Jnk (c-Jun N-terminal kinase), SCF (stem cell factor), LEP (leptin), LHB (luteinizing hormone beta polypeptide), Mapk (mitogen-activated protein kinase), NFkB (complex) (nuclear factor of kappa light polypeptide gene enhancer in B-cells), NOX3 (NADPH oxidase 3), P38 MAPK (P38 mitogen-activated protein kinase), PRKAA1 (protein kinase, AMP-activated, alpha 1 catalytic subunit), PRKAA2 (protein kinase, AMP-activated, alpha 2 catalytic subunit), PSMD4 (proteasome (prosome, macropain) 26S subunit, non-ATPase, 4), SMPD2 (sphingomyelin phosphodiesterase 2, neutral membrane (neutral sphingomyelinase)), TFF3 (trefoil factor 3), AGT (angiotensinogen), APOA1 (apolipoprotein AI), COL18A1 (collagen, type XVIII, alpha 1), CXCL11 (chemokine (C-X-C motif) ligand 11), BLC (B lymphocyte chemoattractant), MIG (monokine induced by gamma interferon), Fcgr2 (Fc gamma R2), FDX1 (ferredoxin 1), FDXR (ferredoxin reductase), FSH (follicle stimulation hormone), GCLC (glutamate-cysteine ligase, catalytic subunit), GH (growth hormone), GHR (growth hormone receptor), SGOT (serum glutamic oxaloacetic transaminase), KIM-1 (kidney injury molecule 1), HSD3B2 (hydroxy-delta-5-steroid dehydrogenase, 3 beta- and steroid delta-isomerase 2), IL6 (interleukin-6), ITK (IL2-inducible T-cell kinase), LDLR (low density lipoprotein receptor), LPL (lipoprotein lipase), LSS (lanosterol synthase (2,3-oxidosqualene-lanosterol cyclase)), MSMO1 (methylsterol monooxygenase 1), NLRP12 (NLR family, pyrin domain containing 12), NPPB (brain natriuretic peptide), NR1H4 (nuclear receptor subfamily 1, group H, member 4), OSMR (oncostatin M receptor), PI3K (complex) (phosphoinositide-3-kinase), SCARB1 (scavenger receptor class B, member 1), TBG (thyroxine binding globulin), SST (somatostatin), Stat5a/b (signal transducer and activator of transcription a/b), TCR (T-cell receptor), TNF-alpha (tumor necrosis factor-alpha), TNFRSF11B (tumor necrosis factor receptor superfamily, member 11b), VWF (von Willebrand factor).

**Figure 3** shows the two top networks from Ingenuity Pathway Knowledgebase software for the group of molecules associated with fatty acid metabolism and hormone function. Molecules are coloured according to typical sex differences and circled where significant interactions were found for Asperger syndrome and sex in [34]. Stem cell factor (SCF) and receptor for advanced glycosylation end products (RAGE) in one network, and growth hormone (GH) in the other network showed significant sex-specific alterations in Asperger syndrome. The software showed that these two networks, containing most of the molecules in the cluster, were partially overlapping.

## Discussion

This is the first large-scale study investigating molecular sex differences in serum of typical individuals. The present findings provide insight into biological pathways with specific differences in male and female participants, significantly extending current knowledge of sex-specific molecular profiles. These naturally occurring differences provide a baseline for comparison against diseases and may help to uncover pathways involved in disease-related sex dimorphisms. A particular strength of the present study was the multiplexed investigation of a large number of molecules covering multiple biological pathways. This allowed a comprehensive assessment of changes in these pathways at the time of sampling and circumvents problems associated with combining results from single molecular assays across studies.

The most significant finding was a higher level of molecules associated with oxidation of fatty acids and hormone function in female participants. It is well known that lipid metabolism differs between males and females, and multiple studies have suggested that these differences are not only a consequence of phenotypical differences such as percentage of body fat, but also related to sex dimorphisms in metabolism itself [35]. In this context, the glycerol rate of appearance, which has been used as an indicator of whole body lipolytic rate, has been found to be higher in women. It has been suggested that the associated increased release of fatty acids can be advantageous under conditions of elevated energy demand, but may also be related to the increased susceptibility of women to develop fatty acid liver disease [35], [36]. It is important to note that participants analyzed in the present study were not matched for percentage of body fat. However, since this is a characteristic that can be expected to differ between males and females in general, it may be an important factor associated with disease or treatment-related sex dimorphisms itself.

The molecules found to be associated with higher fatty acid metabolism in females included adiponectin, leptin and apolipoprotein AI. In contrast, molecules involved in lipid transport, including most of the measured apolipoproteins, showed inconsistent sex differences. To the best of our knowledge, this is the first report showing different effects of sex on lipid metabolism and transport. Elevated lipid metabolic rate coupled with few changes in the levels of many of the apolipoproteins involved in lipid transport in females may reflect intrinsic sex differences in the handling of lipids, including metabolism, transport and storage. For example, a study investigating basal, postabsorptive very low density lipoprotein – triglyceride (VLDL-TG) kinetics identified higher female secretion rates as well as differences in production in clearance [35].

A second important finding was that higher levels of fatty acid metabolism and hormone regulation-related molecules coincided with elevated levels of immune cell chemotaxis proteins in males. This is interesting since molecules related to immune cell growth and activation showed the opposite behaviour and were increased in females.

It has been established that humoral and cell-mediated immune responses are generally more active and robust in females compared to males and that inflammation and production of inflammatory markers show sex differences [1], [37]–[39]. Polymorphonuclear leukocytes (PMNs) in females were found to have vigorous phagocytic responses after anaesthesia, surgery, introduction of lipopolysaccharide, and acute ethanol intoxication [37]. Women also have higher amounts of serum antibodies, CD4^+^ T cells and CD4/CD8 T cell ratios in blood, along with higher cytokine expression during infection and stronger T cell humoral immune responses [40]. The possibility that such differences could lead to disease-related sex dimorphisms is exemplified by evidence that dysregulation of the IL1 agonist/antagonist system may cause greater severity of chronic fatigue syndrome [41]. Also, females have a poorer prognosis in cases of chronic inflammatory conditions such as cystic fibrosis and chronic pulmonary obstructive disease with greater morbidity and complications [38]. Immune reactivity has also been linked to the higher prevalence of autoimmune diseases in females [1], [41].

Recruitment of immune cells to sites of infection and inflammation is necessary to coordinate immune response and is important in disease, as the type of chemokine produced in a specific condition influences inflammation [42]. Though females generally show higher production of cytokines upon infection, we found that analytes related to immune cell chemotaxis and cell signalling were present at higher levels in males. These analytes included several growth factors, monocyte chemotactic proteins and macrophage inflammatory proteins. Lower levels of these molecules in females are consistent with reported effects of estrogen down-regulating chemokine production. Previous studies have shown that estrogen treatment leads to decreased mRNA transcription of chemoattractant proteins in macrophages and increased production of cytokines from immature dendritic cells [41], [43]. Monocytes and macrophages also showed reduced expression of pro-inflammatory cytokines due to the effects of estrogen on receptor CD16 [43]. Overall, the higher levels of many chemotactic factors found in males in this study may be due to a lack of suppressant effects of estrogen at basal levels. This could have important effects, especially in the initial response to infection, autoimmunity, and diseases with links to specific chemokine effects. For example, autoimmune disease severity in females has been linked to fluctuating estrogen levels and the type of cytokine environment that they induce [40].

Differences in cytokines and inflammatory processes have also been linked to alterations in lipid metabolism, providing further support for the co-occurrence of such differences in the present study. Adiponectin has been found previously to be elevated in females and this sex difference decreased linearly with the stage of diabetes progression. The inflammatory molecules C-reactive protein (CRP) and IL1 receptor antagonist (RA) were also increased in pre-diabetic and diabetic women, and these increased even further as the disease progressed, relative to the findings in males [44]. Adiponectin has also been found to decrease endothelial cell CRP synthesis and secretion [45]. Adiponectin and CRP were both found to be present at higher concentrations in females in our study, and were associated with the fatty acid metabolism analyte cluster. Such links between lipid metabolism and inflammatory markers have interesting implications in sex dimorphisms in metabolic syndrome, cardiovascular disease and other related disorders [46].

To evaluate sex dimorphisms deviating from the variation seen in normal controls, we investigated a cohort of participants with Asperger syndrome, which is characterised by a particularly pronounced sex difference in incidence. This analysis led to identification of an interaction in a clustered group of molecules enriched in fatty acid catabolism and hormone regulation functions, indicating a deviation from normal sex variation that affected an entire system of molecules. Interactions for individual molecules within the cluster have previously been shown in Asperger syndrome in [34]. Investigation into other molecules part of the detected networks, which have not been associated with Asperger syndrome as yet, has potential to help explain the sex-specific effects in this cluster of molecules and its biological implications for this condition. It is worthwhile to note that the cytokine alterations that were previously observed at the individual analyte level in the same cohort were not apparent at the pathway level [34]. This may be due to the separation of inflammatory molecules into multiple distinct sets combined with a lack of change seen in other related molecules in the cluster. These results indicate the importance of this set of related molecules, associated most strongly with fatty acid oxidation and hormone function, in the molecular basis of Asperger syndrome. This is particularly interesting since Asperger syndrome has been associated with higher free testosterone levels in female participants and testosterone is known to be a strong regulator of lipid metabolism [47]–[49].

### Conclusions

In summary, we have established that important sex differences exist in human serum using a multiplex immunoassay system. Specifically, we found sex differences in molecules related to metabolism and immune cell function, which may be explored more deeply in the context of disorders associated with sex effects. We have also shown that a cluster of molecules associated with fatty acid metabolism and hormone levels exhibits sex dimorphic differences in a cohort of adults with Asperger syndrome. Such differences indicate that Asperger syndrome, and potentially other autism spectrum conditions, may develop differently in males and females according to sex-specific molecular pathways [21], [50]. These findings suggest that there is considerable scope for further studies of the effect of sex on disease susceptibility and development.

## Supporting Information

Table S1 Clusters of molecules. Assignment of all molecules in cohort 7 using k-means clustering.
(XLSX)
